# Neonatal Sex Assignment in Disorders of Sex Development: A Philosophical Introspection

**DOI:** 10.21699/jns.v6i3.604

**Published:** 2017-08-10

**Authors:** V. Raveenthiran

**Affiliations:** Department of Pediatric Surgery, Sri Ramasamy Memorial (SRM) Medical College SRM University, Chennai, India

**Keywords:** Disorders of sex development, Intersex, Ambiguous genitalia, Congenital adrenal hyperplasia, Androgen insensitivity syndrome, Gonadal dysgenesis, Gender dysphoria, Sex reassignment

## Abstract

Management of ambiguous genitalia is highly controversial. This condition was known previously as intersex and presently as disorders of sex development (DSD). There is no consensus regarding the choice, timing and method of sex assignment in neonates with DSD. Consensus conferences could not unify the views of various stakeholders and third parties. This article philosophically examines the nature and origin of such controversies. Misconception, bias and conflicting priorities are identified as the three cardinal sources of controversies. Conceptual duality of sexes, confused notion of sex and gender, bias towards penetrative intercourse, conflict between utopian ideals and reality, unwillingness to compromise are identified as perpetuators of controversies. Suggestions are made regarding sex assignment in various types of DSD based on the understanding of published literature and the author’s personal experience.

**INTRODUCTION**

Sex of a newborn is typically assigned at birth on the basis of genital appearance. Therefore, children with ambiguous genitalia frequently require reassignment of sex either because of incorrect original labeling or because of subjective dissatisfaction with the sex of rearing (gender dysphoria).[1,2] Since sex is a fundamental attribute of human life its reversal after original assignment is fraught with emotional, social and existential turmoil.[3,4] As many as 65% of parents required psychological support at diagnosis.[5] Consequently considerable disagreement exists regarding the choice, timing and method of sex assignment.[6,7] Biological complexity of the issue is further complicated by the involvement of advocacy groups and sensationalizing media.[8] These third parties vociferously accuse physicians guilty of genital mutilation and human rights violations. Doctors have even been sued for alleged impropriety of sex reassignments.[9,10] This volatile atmosphere was feared to adversely affect the wellbeing of affected individuals. Therefore, in 2005, a consensus conference was organized in Chicago to unify the views of various stakeholders.[11] Paradigm shift of the conference was emphasis on sex assignment based on genetic and molecular criteria rather than gonadal function.[12] Old terms such as hermaphroditism, intersex and ambiguous sex were discarded in favour of the newly proposed nomenclature “disorders of sex development (DSD)”. Unfortunately, even after a decade of consensus statement, controversies refuse to die down.[13,14] Focusing only on the controversies rather than their origin could be responsible for this vexatious situation.

Misconception, bias and conflicting priorities are the three cardinal pillars of any controversy. Sex, being a taboo subject, has no dearth of this evil combination. Complexity of sex reassignment can be better understood if approached in the light of these 3 perpetuating factors. This article is intended to philosophically examine the origin, factuality and possible solutions of controversies pertinent to the management of ambiguous genitalia in newborns.

**Misconceived Duality of sexes**

The fundamental flaw of sex assignment is the conceptual duality of sexes. In fact sex of an individual is determined by a conglomeration of factors such as chromosomal pattern (XX vs. XY), nature of gonads (ovary vs. testis), predominance of circulating sex hormones (estrogen vs. androgen), topographic anatomy of genitalia and secondary sexual characters.[15] Usually genital appearance and phenotype are influenced by sex hormones secreted from gonads which in turn are genetically programmed by chromosomal arrangement.[16] Therefore harmony between various determinants of sex is presumed and individuals are neatly categorized into male or female. Problem arises when there is discordance between the various factors. For example, in complete androgen insensitivity syndrome (CAIS) the individual is chromosomally a male with 46XY and has bilateral testes which secrete androgen; but due to receptor deficiency circulating testosterone fails to effect male phenotype. Consequently the individual will externally look like a female with fully developed breasts and labial folds.[17] Contrastingly, in congenital adrenal hyperplasia (CAH) the individual is genetically a female with 46XX and gonads are typically ovaries; but due to deficiency of steroidogenic enzymes excess testosterone is produced thereby leading to virilization. Therefore, girls with CAH will have fused labia mimicking scrotum and hypertrophied clitoris mimicking penis.[18]. Permutation of sex determining factors (Table 1) suggests that sex is a spectrum rather than two neatly packed compartments.[19] Conventional male and female are at the extremes of the spectrum with innumerable shades of sexes lying between them. Surgical reduction of the enlarged clitoris in CAH and excision of testes in CAIS are basically attempts of trimming the individual to suit one of the two artificial categories.

**Figure:**
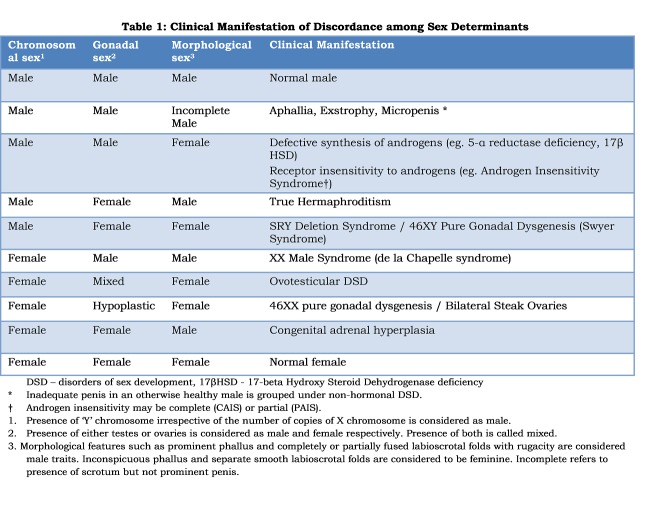
Table 1

**Conflicts of recognizing sex as a spectrum**

Growing voices emphasize recognition of individuals as they are. Although this viewpoint is logically and scientifically ideal, it presents enormous conflicts with established social and ethical principles. Recognizing sex as a spectrum will result in chaos. For example, women upliftment programs will face serious setback because of the overlapping definition of females among various shades of sexes. Disrupting the smooth social order of the majority is as equally unethical as neglecting the needs of DSD individuals. A possible compromise of the conflict is to group all intermediary sexes under “third gender”.[20] Even the Supreme court of India has recently promulgated the constitutional rights of ‘third gender’.[21] However this concept of third gender may not be rational. Homogeneity of components is a prerequisite of defining a group. It may not be logically tenable to include diagonally opposite conditions such as CAH and CAIS under the same umbrella of ‘third gender’. Inclusion of transgenders under this third group adds to the confusion as their problems are very different from that of DSD patients. Therefore, until a radical shift occurs in the societal thinking, rigid compartmentalization of sexes as male and female is indispensable. The conflict between societal outlook and individual preference is best resolved by personalizing the decision of sex reassignment irrespective of the underlying DSD. For example, CAH patients may be assigned to either male or female sex depending upon their individual psychosexual inclination and social circumstances. However genital appearance is no guide to decide the sex of rearing. (Figure 1)

**Figure:**
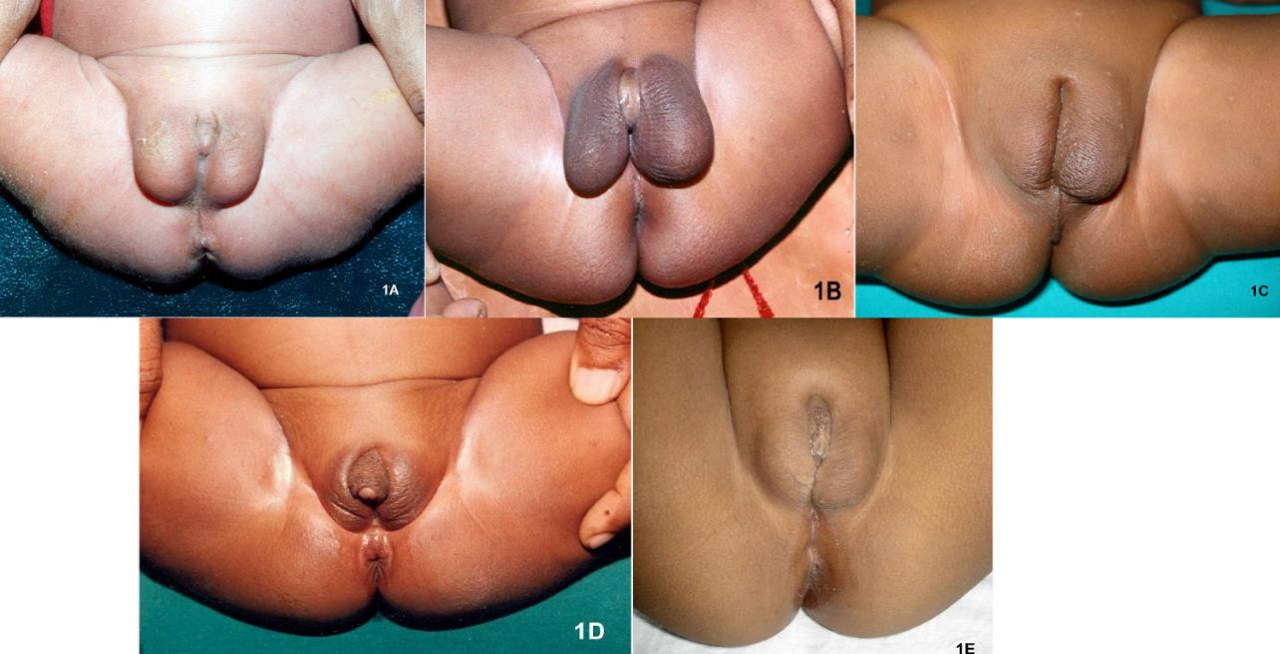
Figure 1: Appearance of external genitalia is no guide to assignment of sex in neonates. Despite similar appearance of the external genitalia the diagnosis and management in each of them are significantly different. (1A). 5-alpha reductase deficiency (46XY, bilateral testes) requires male assignment as the penis will enlarge at puberty; (1B) Severe hypospadias is otherwise an established male in all aspects; (1C). High risk of cancer in mixed gonadal dysgenesis (left testis and right ovary) requires early gonadectomy and female sex assignment; (1D). Congenital adrenal hyperplasia (46XX, bilateral ovaries) is usually assigned to female sex but severe androgen imprinting may require male assignment; (1E). Complete androgen insensitivity syndrome (46XY, bilateral testes) requires female sex assignment due to poor androgenization of body and brain; gonadectomy in them may be postponed until puberty to facilitate development of secondary sexual characters.

**Misconception: Sex versus gender**

Philosophically an individual is made-up of body (soma) and mind (psyche). As Harry Benjamin succinctly put it, ‘Sex is what you see and gender is what you feel’.[22] Both sex and gender are usually concordant in majority of individuals. For example, men behave manly and are attracted towards women while its converse is true of women. This implies that male and female brain must be functioning differently.[23] The greatest blow to the understanding of DSD came when feminists, in their enthusiasm to establish equality of sexes, denied this difference of brain functioning.[24,25] John Money’s theory of gender neutrality at birth[26] indirectly endorsed the feminist view of equality. Interestingly his theory became popular in 1960’s coinciding with the second wave of feminism. According to him both boys and girls are born without any predilection towards social or sexual role play and their subsequent gender-specific behavior is purely determined by social nurturing. Overwhelming importance given to nurture over nature led to bizarre sex reassignments. Boys with aphallia, micropenis and exstrophy were castrated and feminized [27-30] citing Money’s theory as excuse.

Evidence for the fallaciousness of Money’s theory came from his own patient. One David Reimer was born male and he lost his penis in infancy due to a complication of circumcision. Money, confident of his nurture theory, advised him to be brought up as female. Reimer who underwent feminizing genitoplasty was followed up by Milton Diamond.[31] During adolescence Reimer increasingly felt uncomfortable to identify himself as female and he opted for sex reversal operation. Thus nature is proved to prevail over nurture.

The exact mechanism as to how the nature determines gender is poorly understood.[32] Preliminary evidences suggest that fetal brain is masculinized by prenatal exposure to androgen.[33] Peak testosterone levels between 8 to 21 week of gestation appears to facilitate androgen imprinting of male fetal brain.[34] Although androgenization of body and brain often concur, discordance is not unknown. For example, failed or defective androgen imprinting of brain could probably explain homosexuality in an otherwise healthy male.[35] Drawing analogy from this hypothesis, high levels of circulating testosterone is believed to cause varying degree of androgen imprinting in CAH.[36] Thus CAH women with fully virilized brain will have male sexual orientation (homosexual attraction towards females) while those with poor androgen imprinting retain their feminine inclination (heterosexual attraction towards males).[36] Using a single yardstick for sex reassignment in these subgroups will not only be inappropriate but also disastrous.

Sex assignment is relatively easy when sex and gender are congruent than when they are discordant with each other. For example, CAH females with fully developed “penis-like” clitoris and male sexual orientation can be assigned to male sex. But those with slightly prominent clitoris but strongly androgenized brain or vice versa will pose severe dilemma.[37] Hindu philosophy appears to have the solution for this puzzle. In Hinduism ‘atman’ (soul or psyche) is considered superior to ‘sarira’ (body). Psychosexual orientation rather than bodily anatomy should prevail in sex reassignments. Contended mind may adjust with defective body while a healthy body is unlikely to cope up with resentful mind. In essence, sex should be tailored to suit gender.

**Bias towards penetrative sexual intercourse**

Surgical alteration of external genitalia to suit the assigned gender is frequently biased towards feminization. Reconstructing a penis with erectile capacity is technically more challenging than creating a receptive vagina.[38] Popularity of neo-vaginoplasty over neo-phalloplasty indirectly influences sex reassignment. For example, male neonates with inadequate penis such as congenital aphallia, exstrophy, traumatic penile loss and micropenis are often (erroneously) assigned to female sex irrespective of their genetic makeup and gonadal function. On the other hand, enlarged clitoris encroaching upon the vaginal inlet is either resected or reduced in CAH patients. These approaches probably reflect our unconscious bias towards penetrative intercourse.

Evolutionarily sex is intended to be penetrative for sperm transfer and reproduction. However, mankind has moved far from evolutionary purposes. As Masters and Johnson [39] remarked, sex is now intended not only for reproduction but also for recreation and relationship. For the latter two functions vaginal penetration is not essential. In fact, alternate sexual behaviors such as masturbation and oral sex are as equally enjoyable as penetrative sex.[39] Therefore, an intact albeit inadequate genitalia is probably better than an insensate sex organ. Orgasmic difficulty of DSD patients is often attributed to neonatal operative injury of genital nerves. As much as 39% of CAH patients reported orgasmic difficulty despite clitoris preserving genitoplasty. On the other hand 100% those who did not undergo any genital operation reported satisfactory orgasm.[40] These findings suggests that neonatal genitoplasty should be aimed to provide sensually enjoyable organs rather than cosmetically acceptable genitals.

**Conflict between utopian ideals and reality**

Timing of sex reassignment is a highly contentious issue.[41-43] It is caught between the utopian ideals of allowing affected individuals to decide for themselves at puberty and the practical problems of raising these children with gender uncertainty.[44] Long periods of indecisiveness is feared to leave them with confused gender identity and social ridicule.[45,46] Initial gender of rearing is found to be a better predictor of adulthood gender identity and contentedness.[47,48] However, the greatest hurdle is the inability of neonates and infants to express their psychosexual orientations.

Ability to predict future sexuality of infants is an interesting proposition to solve the problem of early sex assignment in DSD individuals. It is suggested that the degree of androgen imprinting of brain and hence a future male inclination can be predicted to some extent from genital appearance and toy preference. (Figure 2) For example, CAIS patients with fully feminized genitalia are more often satisfied with female sex assignment.[49-50] Insensitivity of cerebral androgen receptors could be a logical explanation of this. In CAH, most of those with Prader 4 and 5 type (fully virilized) genitalia are more dissatisfied with female sex assignment than those with lesser Prader-score (partly virilized genitals) although both the group develop unambiguous female identity if the gender is assigned before 24 months of age.[48,51,52] (Figure 2)

Significant difference in the toy preference of boys and girls is thought to correlate with androgen imprinting and future psychosexual orientation.[53] A similar observation of gender-specific toy selection in primates implies that the phenomenon is probably a biological characteristics rather than a mere effect of parental rearing.[54] Therefore it is suggested that children who prefer male-type toys may be assigned to male sex and vice versa. However our current understanding of sexuality prediction is far from completeness. Genital appearance and androgen levels were found to correlate well with gender specific social role play but not sexual orientation.[47] Similarly, gender-specific toy preference is well correlated with gender identity but not with sex role-play.[55] It is well known that gender identity is different from sexuality.[56] CAH girls who may behave boyish may still be feminine in their sexual outlook. From these observations it appears that the degree of androgen imprinting of brain may not be uniform; probably it differs not only between individuals but also between different areas of the brain in the same individual. Further research is needed to test this hypothesis. Until then who should be responsible for decision making on behalf of DSD neonates is the looming question.

In many other spheres of life such as choice of school education, food and vaccination parents take surrogate decision in the best interest of their offspring. Therefore it may not be inappropriate for parents to decide upon the sex of their children when it is ambiguous. However, problems arise when parents take decision under social pressure, misconception, bias or ignorance. For example, in developing countries like India, social stigma is more for an inadequate female than for a deficient male. Sexually handicapped male may still earn a livelihood, can effectively evade sexual abuse and can openly experiment with his sexuality; but the same is not true of DSD neonates raised as females. Therefore, many parents request male sex assignment in CAH despite knowing the possibility of fertility if the child is raised as female.[57] More intriguingly male sex assignment is requested even in CAIS despite acknowledging the futility of such decision. Parental health education, psychosocial support and governmental welfare schemes may mitigate such inappropriate decisions by parent.

**Figure:**
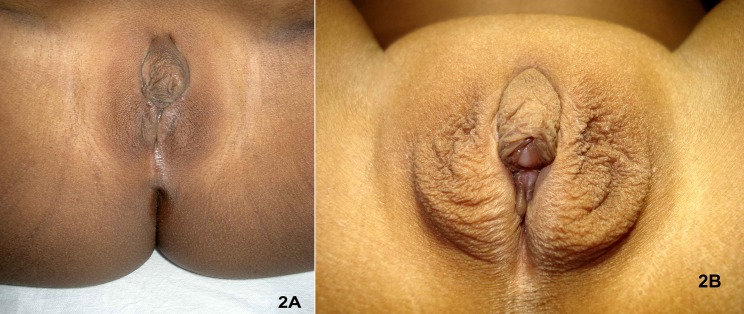
Figure 2: Sex assignment may differ in the same condition according to the degree of cerebral androgen imprinting. Degree of virilization of external genitalia may, but not necessarily, predict the degree of androgen imprinting of brain. (2A) Congenital adrenal hyperplasia (CAH) with minimal virilization (Prader score 2) may predict poor androgen imprinting and hence female assignment is appropriate. (2B). CAH with severe virilization (Prader score 4) are often dissatisfied with female sex assignment. It is probably due to strong androgen imprinting and hence male assignment is appropriate in this subset.

**Conflict between procreativity, sexuality and sociability**

Gonads are meant for procreation while external genitalia offers sexual pleasure and concordant secondary sexual characters enhance social interaction. Achieving harmony between all the three components is a utopian ideal desired in every DSD neonates; [58] but the harsh reality necessitates sacrificing one or two of them to achieve successful outcome. For example, retained testes of partial androgen insensitivity syndrome (PAIS) males may facilitate future procreation by assisted reproductive techniques. However mismatched external appearance at adolescence consequent to testosterone secreted from the testes will stigmatize the individual and adversely affect sociability. Prioritizing is easier if the gonads carry high risk of malignancy such as that of streak gonads. [59] In such cases “safety of life” principle negotiates all other ethical dilemma. But gonadectomy is fraught with serious ethical problem when the risk of cancer is low as it is in non-hormonal DSD. When faced with conflicting priority between sexuality and procreativity the former should be given preference over the latter as exemplified by the meaningful life of infertile couple who are otherwise healthy. Research data indicate that DSD patients with social acceptability and career success are satisfied with their gender of rearing irrespective of sexual satisfaction or procreative ability.[60] Success achieved by sacrificing a few is more endurable than failure resulting from attempted preservation of all.

**Conclusion**

Acknowledging that utopian ideals are different from reality is an essential prerequisite of resolving the controversies of sex reassignment. Willingness to compromise in case of competing priorities, elimination of misconceptions and overcoming bias are necessary adjuncts. Sex reassignment should be personalized for each patient irrespective of the underlying disease. Physical appearance should be tailored to suit psychosexual orientation. When it is impossible to know the mental inclination of neonates and infants, informed decision of parents should prevail. Prolonged uncertainty of gender is better avoided and early sex assignment is recommended although it may be far from ideals. Sex assignment should be aimed to preserve sociability, sexual satisfaction and procreative ability in that order of importance. Genitoplasty should be aimed to provide sensually enjoyable organs rather than cosmetically acceptable genitalia. Further research on the nature of androgen imprinting of brain and ability to predict it in infancy may add more clarity to the understanding of gender development and sex assignment. Nevertheless, interference of social activism with medical science will be detrimental for elucidation of truth.

## Footnotes

**Source of Support:** None

**Conflict of Interest:** None

**Disclaimer:**Views expressed in this article reflect the author’s understanding of neonatal sex assignment rather than any official recommendation.
